# Food allergy in mice is modulated through the thymic stromal lymphopoietin pathway

**DOI:** 10.1186/s13601-016-0090-2

**Published:** 2016-01-19

**Authors:** Christophe P. Frossard, Simone C. Zimmerli, José M. Rincon Garriz, Philippe A. Eigenmann

**Affiliations:** 1 Inflammation and Allergy Research Group and Department of Pediatrics, University Hospitals of Geneva and University of Geneva, 6 rue Willy-Donzé, 1211 Geneva 14, Switzerland; 2 Allergy Unit, University Hospitals of Geneva and University of Geneva, Geneva, Switzerland; 3 EMD Serono, Billerica, MA USA; 4 Fasteris SA, Plan-les-Ouates, Switzerland

## Abstract

**Background:**

Thymic stromal lymphopoietin (TSLP) is involved in the pathogenesis of allergic reactions in the skin and the lung. Nevertheless, data on the role of TSLP in food allergy are scarce. We explored the role of TSLP in a mouse model with oral sensitization and oral challenge eliciting food allergy.

**Methods:**

TSLP receptor (TSLPR)−/− mice and wild type mice were orally sensitized to β-lactoglobulin in presence of cholera toxin (CT) or CT alone. The elicited immune response was characterized in vitro and the mice were subsequently challenged with the antigen. Lymphocytes from various locations in the gut were activated either by the antigen or by CT and assayed for cytokine secretion.

**Results:**

Here we report that TSLPR−/− are less prone to generate food-induced reactions in conjunction with a decreased antigen-specific IgG1, but not IgE response. In addition, mesenteric lymphnode lymphocytes of TSLPR−/− mice were secreting lower quantities of IL-4, IL-5 and IL-10 after in vivo Ag activation, whereas higher numbers of IL-17 secreting cells were observed. Similarly, activation by the Th2-type adjuvant cholera toxin resulted in an increased frequency of IL-12 and IL-17 secreting lamina propria and mesenteric lymphocytes, together with increased production of IL-12 by activated dendritic cells in TSLPR−/− mice.

**Conclusions:**

TSLP can be considered as an essential, but not exclusive, mediator for elicitation of food allergy in mice, as well as a potential target for future therapeutic interventions.

## Background

The pathogenesis of food allergy involves various mechanisms, all closely associated with the gut-related immune system [1]. Initiation of IgE-mediated food allergy follows the path of Th2-type sensitization involving antigen presentation and CD4+ T cells, followed by IL-4 and IL-13 facilitated antigen-specific IgE production. Mice models of food allergy with oral sensitization to common food antigens eliciting anaphylactic reactions upon re-exposure have allowed extensive description of Th2-type, gut-related mechanisms of IgE-mediated food allergy [2, 3]. In addition to IgE-dependent pathways of gut-mediated anaphylaxis, other mechanistic pathways have been described, e.g. by involving IgG1 antibodies, or antigen-activated complement [4, 5]. Thymic stromal lymphopoietin (TSLP) dependant mechanisms of food allergy have also been suspected [6, 7].

TSLP has a close four helix structural analogy to IL-7, and can be found secreted in increased amounts in epithelial cells (EC) of the skin, the lung and the gut. TSLP is expressed in presence of the Th2–type cytokines IL-4 and IL-13 [8–13]. TSLP in relation to allergy has been first studied in atopic dermatitis where increased levels have been found in inflamed skin associated with Th2-type cytokines [14]. Similarly, TSLP receptor (TSLPR)^−/−^ mice lacking responsiveness to TSLP fail to express Th2-type cytokines and lung inflammation [15, 16]. It has also been demonstrated that skin-derived dendritic cells are targets of TSLP in the Th2-type immune response in the skin [17]. In the gut, intestinal EC produce TSLP, and expression of TSLP and the TSLPR are closely linked to inflammation mediated by IL-12, IL-17, and Th2-type cytokines [18, 19].

We hypothesize that, similarly to the skin and the lung, IgE-mediated immunity in the gut is regulated by TSLP and its receptor. For these studies, we used a well characterized mouse model with oral antigen sensitized with β-lactoglobulin (BLG) in presence of the Th2-type adjuvant cholera-toxin (CT). The main characteristic of this widely used model are symptoms of anaphylaxis upon food gavage [20, 21], the most closely reproducing symptoms seen in food allergy in humans. Clinical parameters and biomarkers were measured in wild-type (wt) and TSLPR−/− mice in the light of two specific aims: (1) to investigate if a functional TSLPR was instrumental in eliciting food allergy, and (2) to assess the role of CT in relation to a functional TSLPR in the sensitization process.

## Methods

### Mice

BALB/c female mice were purchased from Charles River (L’Arbresle, France) and were housed at the Animal Facilities of the University of Geneva, School of Medicine. TSLPR−/− mice [22] were backcrossed to a BALB/c background for eight generations or more. All animals were used between 4 and 5 weeks of age and were fed with standard mice pellets without milk proteins. All experiments were approved by the Animal Studies Ethics Committee and performed in accordance to their guidelines.

### Antibodies, reagents and medium

Anti-CD11c (HL3), anti-IL-4 (11B11), anti-IL-5 (TRFK5), anti-IL-10 (JES5-16E3), anti-IL-12p70 (C15.6) and anti-IL-17 (TC11-18H10) were from BD Pharmingen (Allschwil, Switzerland). Anti-IL-13 was from eBioscience (eBio13A) (Vienna, Austria).

CT was from List Biological Labs (Campbell, CA, USA). BLG was from Sigma (Buchs, Switzerland).

RPMI 1640 and DMEM medium were supplemented with 100 U/ml penicillin, 100 µg/ml streptomycin, 2 mM l-glutamine, 100 μg/ml gentamicin, 15 mM HEPES pH 7.4 and 10 % heat-inactivated FCS. In addition, DMEM was supplemented 2 × 10^−5^ M 2-mercaptoethanol, 1 % nonessential amino acids and 1 mM sodium pyruvate (all reagents from Sigma).

### In vivo sensitization, and BLG specific challenge

Mice were sensitized 5 times 1 week apart by oral administration of 10 µg of CT in association with 10 mg of antigen in a solution containing 0.2 M of NaHCO_3_, pH 9. An oral antigen challenge was performed 1 week after the last sensitization by gavaging 100 mg BLG. A score based on symptoms of anaphylaxis (*0* no reaction; *1* mild reactions: decreased activity, random scratching, myocloni; *2* moderate reactions: marked decreased activity, continuous scratching, abnormal breathing; *3* severe reactions low or absence of reactivity, abnormal breathing leading occasional to death) was assessed [21]. Mice were thereafter sacrificed at the indicated time and their intestinal lymphoid cells isolated.

### Measurement of BLG-specific antibodies in mouse serum

Sera were obtained 7 days after the last sensitization by tail vein bleeding. BLG-specific Ab titers were measured by a method adapted from Adel-Patient et al. [23] as described previously [24].

### Isolation of lymphocytes

After sacrifice of the mice with CO_2_, mesenteric lymph nodes (MLN) were isolated, the small intestine was excised, and Peyer’s patches (PP) lymphocytes, lamina propria lymphocytes (LPL), intraepithelial lymphocytes (IEL) and epithelial cells (EC) were isolated using modified methods as previously described [25, 26]. Briefly, the small intestine was opened longitudinally and cut into 5-mm pieces. The tissue was incubated in calcium- and magnesium-free cHBSS containing 2 mM EDTA and 1 mM dithiothreitol (Sigma) for 30 min at 37 °C with magnetic stirring. It was then vigorously vortexed and filtered through a 70-μm nylon filter in order to obtain a cell suspension. The cell population was washed twice and separated by discontinuous 33/40 % Percoll (Bioscience, Uppsala, Sweden) on a lympholyte M gradient (Cederlane, Hornby, Canada) for 20 min at 600 g at room temperature. IELs were harvested on the Percoll 40 % lympholyte M interface, and ECs on the 30 % interface.

### Cytokine measurement by ELISPOT

ELISPOT plates were coated with streptavidin overnight at 37 °C, followed by addition of 1 μg of biotinylated BLG for 3 h. Lymphocytes isolated on a Percoll 60/66 % gradient (Amersham, Zurich, Switzerland) were resuspended as 2 and 1 × 10^6^ in Iscove’s modified Dulbeco medium supplemented with penicillin, streptomycin, l-glutamine, 100 μg/mL gentamycin, polymixin B + 5 % FCS for 24 h at 37 °C, followed by overnight incubation at 4 °C with the corresponding antibodies. Amino-ethyl-carbazole 100 μl/well was added for 10 min, and the spots were automatically counted by the KS EliSpot 4.2.1 Software (Zeiss, Halbermoos, Germany) and expressed as cell forming units (CFU) per 10^6^ cells.

### Real-time PCR

Total RNA from PP lymphocytes, LPL, IEL or EC was extracted with Isol-RNA Lysis reagent. 5 x 10^6^ cells were first centrifuged 5 min at 1400×*g* and the pellet was resuspended in 1.0 ml of Isol-RNA Lysis reagent for 5 min., 0.2 ml chloroform was added and tubes were mixed by inversion for 30 s and left for 3 min at room temperature. Samples were then centrifuged for 15 min at 14,000 rpm (4 °C) resulting in a colorless upper aqueous phase that contains the RNA and a lower red organic phase. 0.5 ml isopropyl alcohol was added to the aqueous phase in order to precipitate RNA. After centrifugation, the RNA pellet was washed once in 1 ml 75 % ethanol. At the end of the procedure, the RNA pellet was briefly air-dried and resuspended in 40 μl RNAse-free water.

RNA content of each sample was assessed using a nanodrop ND-1000 spectophotometer. 1 µg of RNA was used to perform the semi-quantitative RT-PCR with specific primers for the mouse TSLP gene. The PCR was realized [using taq DNA polymerase from Invitrogen (Zug, Switzerland)], with the following primers: 5′-GACAGCATGGTTCTTCTCAG-3′ and 5′-CTGGAGATTGACATGAAGG-3′ (30 cycles of 94 °C for 60 s denaturation, 55 °C for 60 s annealing, and 72 °C for 60 s extension). As a control, the same amount of RNA was used to perform a semi-quantitative RT-PCR with specific primers for GAPDH. The final result was expressed as the ratio of the signal obtained for TSLP divided by the signal obtained for GAPDH.

### Statistics analysis

Statistical significance between groups was analyzed by using the Wilcoxon rank-sum test for nonparametric unpaired data. For assessment of clinical reactivity, batches of 4–5 mice were challenged simultaneously, and the experiments were repeated twice. Antibody titers measurements and cell experiments were performed with pooled samples from three mice per experiments, and repeated 3 times.

## Results

### TSLPR−/− mice have less pronounced food allergy symptoms

**Fig. 1:**
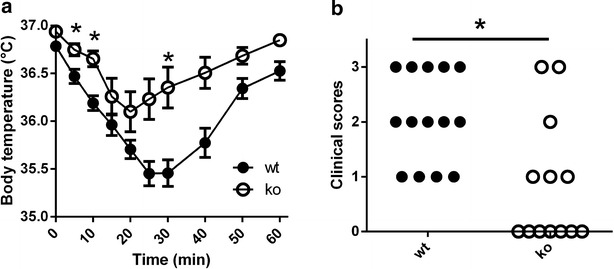
Reduced severity of anaphylactic reaction in CT-sensitized TSLPR−/− mice. BLG-sensitized mice, either wild type (wt) and TSLPR−/− mice (ko) are challenged by oral gavage with 100 mg of BLG 7 days after the last sensitization, the body temperature is recorded on the ear (**a**). In addition symptoms of anaphylaxis were clinically assessed (*0* no reaction to *3* severe reaction) (**b**). The results are expressed as a pool of data from independent experiments. *p < 0.05

In order to generate mice with an allergic phenotype to foods, sets of mice were sensitized 5 times at 1 week interval by intra-gastric gavage with BLG in presence of CT. Allergy was characterized by mean of a subsequent oral BLG challenge. Mice were clinically evaluated by a symptom score published earlier [21]. In order to add an objective measure, the severity of anaphylaxis was correlated to the measure of decreased body temperature. We observed that wt mice were showing symptoms of anaphylaxis in form of decreased activity, swelling of the snout, erythema of the face and feet, as well as itching and erected pilli. Increased breathing and severe reactions leading to death were also observed. In correlation to these observations, marked decreased body temperature was seen, peaking at 30 min after gavage (Fig. 1a). Conversely, assessment of TSLPR−/− mice at the same time points showed predominantly mild or no symptoms of anaphylaxis (Fig. 1b) and a faster recovery correlating to only a slight decrease in body temperature. These results suggest that food-mediated allergic reactions are, to a significant extent, mediated by a TSLP-dependent mechanism.

### How do antigen-specific serum antibody levels correlate with TSLP?

**Fig. 2:**
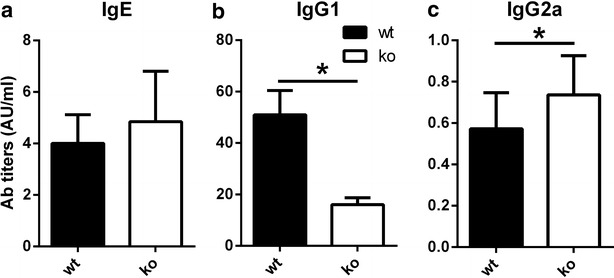
Th2-related and Th1-related Ab profiles in BLG-sensitized wild type and TSLPR−/− mice. Serum IgE (**a**), IgG1 (**b**) and IgG2a (**c**) Abs specific for BLG in BLG-sensitized wild type (wt) and TSLPR−/− mice (ko) 7 days after the last sensitization. The results are expressed as arbitrary units from three independent experiments. *p < 0.05

We were then interested in characterizing BLG-specific antibody production in wt, and TSLPR−/− mice. Serum was collected 7 days after the last oral antigen-sensitization and BLG-specific IgE (Fig. 2a), IgG1 (Fig. 2b) and IgG2a (Fig. 2c) antibody titers were measured by ELISA. Clearly, wt mice had much higher titers of IgG1 (in wt mice: mean 51.0 ± 9.4 AU/ml, TSLPR−/− mice: mean 16.0 ± 2.7 AU/ml), a Th2-type related antibody isotype, while IgG2a, a Th1-type related isotype was increased in TSLPR−/− mice. IgE titers were similar in both groups.

### Is the modulation of Th2-type cytokine secretion after in vivo antigen-specific cell activation TSLPR dependent?

**Fig. 3:**
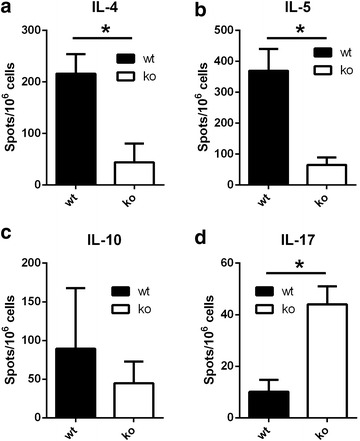
Reduced Th2 cytokines and increase of IL-17 in BLG-sensitized TSLPR−/− mice by ELISPOT. Ex vivo cytokines secretion by mesenteric lymphnode lymphocytes 8 h after oral boost with 20 mg of BLG in NaHCO_3_ in wild type (wt) and TSLPR−/− (ko) mice. The results are expressed as number of cytokines forming spots/10^6^ cells from three independent experiments. *p < 0.05

In order to correlate cytokine production and TSLP expression after sensitization to BLG, we isolated MLN cells 8 h after infra-clinical antigen gavage given for in vivo cell activation and measured their ex vivo cytokine production by ELISPOT. The presence of the TSLPR was clearly modulating cytokine production, as shown by a decreased secretion of IL-4, IL-5 and IL-10 in TSLPR−/− mice (79 % decrease of IL-4 secretion, 80 % of IL-5, 51 % of IL-10; significant for IL-4 and IL-5 but only trend for IL-10) (Fig. 3). In contrast, we did measure strongly increased levels of IL-17 (4.4 times) in TSLPR−/− mice. IL-12 (not shown) was secreted in similar amounts by MLN cells isolated from both type of mice.

### CT activates TSLP in gut epithelial cells

**Fig. 4:**
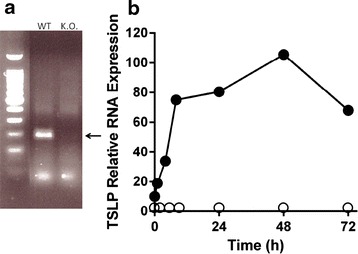
CT induced expression of TSLP in vivo. **a** TSLPR expression in epithelial cells from wild type (WT), and TSLPR−/− mice (K.O.). The band representing TSLP is marked by an* arrow*. **b** Relative TSLP mRNA expression by intestinal epithelial cells from WT mice (*close circles*) and from K.O. mice (*open circles*) after oral administration of 10 mg of cholera toxin. The results (in %) are normalized to GAPDH expression

We next investigated whether TSLP RNA expression was related to the CT administration given by gavage during the sensitization. RNA expression of TSLP by gut EC, Peyer’s patches cells, lamina propria lymphocytes and intra-epithelial lymphocytes from wt mice was measured by semi-quantitative RT-PCR at various time points after CT administration. Only EC (other cells not shown) were found to express TSLP RNA after administration of CT (Fig. 4a). Figure 4b shows the progressively increased expression of TSLP RNA with a peak at 48 h post CT administration, clearly showing a CT-related TSLP RNA expression in gut EC of wt mice.

### Does the TSLPR regulate CT-induced inflammation in the gut?

**Fig. 5:**
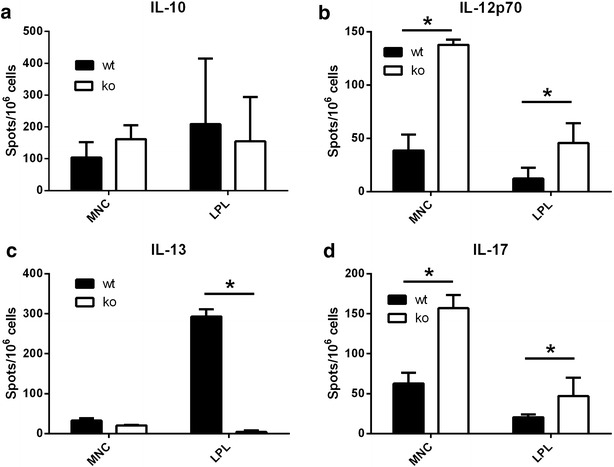
Increased frequency of IL-12 and IL-17 producing cells in CT-sensitized TSLPR−/− mice by ELISPOT. *Ex vivo* cytokine secretion by mesenteric lymphnode (MLN) or lamina propria (LPL) lymphocytes 24 h following oral gavage with 10 μg of CT in NaHCO_3_ in wild type (wt) and TSLPR−/− (ko) mice. The results are expressed as number of cytokines forming spots/10^6^ cells from three independent experiments. *p < 0.05

Next we explored if and to what extent, the TSLPR was regulating CT-induced inflammation. By ELISPOT*, w*e measured ex vivo CT-induced cytokine production of mesenteric lymph node (MLN) cells and lamina propria cells (LPL) from wt and TSLPR−/− mice after oral gavage with CT. Figure 5 shows a clear modulation of IL-12 secretion by TSLP in both MLN and LPL as IL-12 is increased in TSLPR−/− mice (MLN cells in wt mice: mean 38.6 ± 14.9 spots/10^6^ cells, TSLPR−/− mice: mean 137.8 ± 4.9 spots/10^6^ cells; LPL in wt mice: mean 12.2 ± 8.3 spots/10^6^ cells, TSLPR−/− mice: mean 45.7 ± 15.3 spots/10^6^ cells). Similarly, IL-17 expression was strongly increased in TSLPR−/− mice (MLN in wt mice: mean 62.8 ± 13.4 spots/10^6^ cells, TSLPR−/− mice: mean 156.9 ± 16.5 spots/10^6^ cells; LPL in wt mice: mean 20.5 ± 2.9 spots/10^6^ cells, TSLPR−/− mice: mean 47.0 ± 18.8 spots/10^6^ cells). IL-13 secretion was strongly decreased in TSLPR−/− mice, but only in LPL. IL-10 secretion was not influenced by the absence of expression of the TSLP receptor. These results are suggesting that IL-12 and IL-17-driven inflammation in the current model is regulated by TSLP in the gut.

## Discussion

Experiments presented here clearly show that food allergy in mice induced after oral antigen sensitization in presence of CT followed by an oral antigen challenge is strongly, but not exclusively, dependent on a functional TSLP pathway. Absence of TSLP receptor expression in knock-out mice biases antigen and CT sensitization towards a reduced Th2/increased Th17-type profile, and influences dendritic cells to promote a Th1-type antigen presentation. TSLP can be considered as a protein contributing to modulating food allergy in the mouse model described here.

The pathogenesis of immediate-type food allergy is strongly Th2-type oriented with subsequent IgE production and IgE-mediated immediate-type symptoms. We and others have previously described that intestinal T cells produce Th2-type cytokines, and that the gut-associated lymphoid structures are strongly involved in the pathogenesis of the disease [2, 3]. Nevertheless, IgE-independent mechanisms have also been described, involving IgG antibody isotype or complement-driven inflammation [4, 5]. TSLP in the context of allergy has so far been more extensively studied in atopic dermatitis and asthma. In these diseases, it has been shown that in the skin TSLP is essential for eliciting a Th2-type skin sensitivity, as TSLPR−/− mice were unable to mount a Th2-type cytokine production, with the resulting absence of antigen-specific IgE production, and of skin inflammation upon re-exposure to the antigen [17]. In the experiments we report here, we were able to show similar results in the gut. We observed that not only TSLP is essential for the secretion of the Th2-type cytokines IL-4 and IL-5, but also that the Th1 and Th17 pathways are TSLP dependent, as IL-12 and IL-17 were strongly increased in TSLPR−/− mice. These results are in line with previous publications which have shown increased Th1 and Th17 responses in TSLPR−/− mice [18, 19]. In this regard, we have not seen a modified IgE response in TSLPR−/− mice, while IgG1 was significantly lower in TSLPR−/− mice. This finding is correlated to previous studies reporting that in mice mechanisms independent of IgE might be involved in the pathogenesis of food allergy [27], and suggests that TSLP might be linked to them. It is also well known in humans that sensitization to IgE might appear independently of clinical food allergy [28].

Gut intraepithelial lymphocytes constitutively express IL-17 [26], and it has been clearly shown that this cytokine is involved in inflammation in the gut. In addition, it has been shown that TSLP expression and IL-17A is linked, as disruption of the TSLP-TSLPR pathway resulted in increased expression of IL-17A, and the development of severe gut inflammation [19]. In relation to food allergy, the study by DeLong et al. reports activation with the major peanut allergen Ara h 1 of T cell-lines and clones from peanut allergic subjects. While all cells produced IL-4, IL-17 secreting cells were also found. Interestingly, the percentage of cells co-producing IL-4 and IL-17 was minimal, suggesting similarly to our study an antagonistic role for IL-4 and IL-17 [29]. Decreased IL-17 secretion has also been observed in antigen-activated CD4+ cells from food allergic children when compared to tolerant controls [30]. This suggests that in humans, IL-17 may be involved to some extent in the regulation of food allergy. Similarly to food allergy, in eczema and asthma the paradigm of the dysbalance of Th1 and Th2 cells has been completed by increased knowledge of the role of Th17, as well as other T cell sub-types [31].

The role of TSLP has been ascertained in the regulation of various experimental types of allergy, e.g. in models of skin allergy, such as atopic dermatitis [32] or nickel allergy [33], or in allergic airway diseases [15, 16]. Similar observations have been made in human studies [14, 34]. Several investigations addressed the role of TSLP in food allergy. Hong et al. have suspected a role for TSLP, as a specific single nucleotide polymorphism of the TSLP gene was correlated with an increased sensitization to common food allergens in correlation to breastfeeding [35]. Also, eosinophilic esophagitis, an inflammatory disease of the esophagus associated with food allergy characterized by esophageal eosinophilia, has been associated with TSLP gene polymorphisms [36]. Links between the skin and the gut have also been explored. TSLP-associated antigen sensitization through the skin has been shown to induce diarrhea and anaphylaxis after oral re-exposure to the same antigen [37]. Also, TSLP has been found to modulate allergy through antigen exposure on the skin and subsequent allergic reactions similar to our model [6]. In the gut, TSLP is also produced by DCs, and protects against inflammatory colitis [38]. Exploring the role of TSLP in two separate murine food allergy models, Blazquez et al. have found that allergen-induced diarrhea was TSLPR dependent, while the Th2-type sensitization model they used was TSLP independent with similar antigen-specific IgE titers, and absence of decreased anaphylaxis upon allergen reexposure [7]. However, in their studies mice were challenged by i.p. injections, while we gavaged them, this can possibly explain the difference in the antigen-induced anaphylaxis score. Also, antigen-specific IgG1 titers were not measured, and an IgE-independent mechanism cannot be excluded. Chu et al. have observed that after a similar oral sensitization protocol than the one we used, but followed by an i.p. antigen challenge, the procedure elicited a similar anaphylactic response in TSLPR−/− mice than in wt mice [39]. In these studies, besides IgE IgG1 titers were measured and both were found similar in wild type and TSLPR−/− mice. The results in these two studies differ from ours.

Our results suggest that absence of a functional TSLP does only partially prevent food-induced sensitization, as TSLPR−/− mice are able to mount an antigen-specific Ab response. In addition, absence of functional TSLP does not prevent a response to the strong stimulus of i.p. food antigen challenge, while it significantly reduces the anaphylactic response after a more physiological gastric antigen challenge. Altogether, these results suggest that TSLP, in addition to other factors, intervenes in the pathogenesis of food allergy in the mouse models studied here.

It has been well recognized that allergy in general and food allergy in particular is mediated by several, complementary, but independent pathways. Options for treatment or prevention have targeted several of these pathways. In various studies, therapeutic options including oral administration of IL-12 [40], oral administration of a synthetic agonist of Toll-like receptor 9 [41], or blockade of platelet-activating factor and histamine [42] have been investigated for treatment of food allergy. Recently, a preliminary trial in patients with allergen-induced asthma and airway inflammation has found a therapeutic value for an anti-TSLP monoclonal immunoglobulin [43]. Here we define TSLP as a new target for modulation of food allergy. Indeed, we identified TSLP as an essential contributor to the pathogenesis of the Th2-type inflammation elicited after oral sensitization followed by an oral challenge. The immunological parameters correlated with a strong decrease of clinical reactivity upon oral antigen challenge reinforcing a strong dependency of food allergy on TSLP. Defining whether this is due to a lack of direct control of TSLP on T cells or on DCs will need further exploration.

## Conclusions

In conclusion, in the set of experiments presented here, we show that food allergy in mice is in part dependent on a functional TSLP receptor, as antigen sensitization in TSLPR−/− mice was biased towards an allergy protective, reduced Th2/increased Th17-type immune response with decreased clinical reactivity. New therapeutic strategies might consider including TSLP as a target for modulation of food allergy.
